# A comparative study of the curriculum in master degree of medical education in Iran and some selected countries

**DOI:** 10.1186/s12909-023-04366-2

**Published:** 2023-05-30

**Authors:** Somayeh Akbari Farmad, Ali Esfidani, Sara Shahbazi

**Affiliations:** 1 Department of Medical Education, Virtual School of Medical Education and Management, Shahid Beheshti University of Medical Sciences, Tehran, Iran; 2 Department of Medical Education, School of Medicine, Mashhad University of Medical Sciences, Mashhad, Iran; 3 Community-Oriented Nursing Midwifery Research Center, Shahrekord University of Medical Sciences, Shahrekord, Iran; 4 Virtual School of Medical Education and Management, Shahid Beheshty University of Medical Sciences, Tehran, Iran

**Keywords:** Comparative study, Curriculum, Master degree, Field, Medical education, Iran, School Admission Criteria, Universities, Students

## Abstract

**Background:**

When training efficient human resources for the health system, it is necessary to train appropriate student teaching and assessment methods and necessary skills for educational planning and evaluation. Therefore, studies and efforts to train human resources in the field of Iranian medical education have begun since 1994. The aim of the present study is a comparative study of the curriculum in master’s degree of medical education in Iran and some selected countries.

**Methods:**

This is applied, descriptive and comparative research. Data were collected by electronic search on the website of the selected universities. Each of these selected educational curricula, the newest curriculum of the studied universities at the time of the present research, was translated into fluent Persian and studied in detail. The model used in the present study is the famous Polish Bereday model. A quota sampling method was used and universities were selected at a 1:10 ratio from each classified area. Institutions that offered master’s degrees in medical education were chosen in each region using the World Health Organization (WHO) classification based on QS World University Rankings (2020). The data collection instrument was a researcher-made checklist, which was used to extract the relevant data available on the website of selected universities. This checklist consisted of eight items, which included course title, course length, mission, vision, goals, admission process, teaching methods (online, In-person, and both), educational strategies, teaching methods, and student assessment. These eight items were compared at selected universities.

**Results:**

The samples included seven selected universities including Kebangsaan University in Asia, the University of Toronto in Canada, the University of Michigan in America, the University of Bern and Imperial College in Europe, Monash University in Australia, and Iranian universities. Student admission in Iran is carried out through a centralized exam; therefore, most faculties do not have the option to select students and criteria for student selection. The course length in all universities is between 1–3 years (depending on part-time/full-time) and most of the studied universities offer this field as modular degree courses.

**Conclusions:**

The characteristics of the curriculum for the master’s degree of medical education in Iran and selected countries showed the differences and similarities of this course among the top universities of different continents. Unlike other countries, the curriculum for a master’s degree in medical education in Iran is offered in a centralized manner in eight universities of Tehran, Shahid Beheshti, Iran, Isfahan, Shiraz, Tabriz, Kerman, and Mashhad.

## Background

Sustainable and comprehensive development of the higher education system requires balanced growth in quantitative and qualitative dimensions. While qualitative dimensions are usually considered less frequently due to their greater complexity. Paying attention to quantitative growth and neglecting qualitative growth imposes adverse consequences on this system such as academic failure, academic dependence, and lack of creativity and entrepreneurship, brain drain and poor academic achievements. The educational system is of great importance when training specialized human resources that can accelerate the movement towards public development [1]. Universities and higher education institutions, as implementers of educational systems, must respond to the enormous changes that are taking place around them and be able to compete effectively in the evaluation field, especially international evaluation, and quality assurance at the global level [2].

In this regard, there is an increasing trend in medical fields today, and medical universities, in addition to providing healthcare services, have an important mission, that is, training capable and competent people who have the necessary knowledge, attitude and skills to maintain and improve the public health status [3, 4]. When training efficient human resources for the health system, it is necessary to teach appropriate teaching and student assessment methods and train specialists mastered the necessary skills for educational planning and evaluation. Therefore, studies and efforts to train human resources in the field of medical education in Iran have begun since 1994 [5, 6].

Therefore, the curriculum in master degree and Ph.D. of medical education was approved by the High Council for Medical Science Planning in 1994 and 2007, respectively. The revised master’s degree curriculum was approved by this council in 2010, and has been running in medical universities since October 2011 [6].

Investigating the curriculum in master degree of medical education showed that there were only seven curricula at the master’s level in health professional education until 1996, but there are now 76 universities that included this field in their curriculum and presented it under different titles that include:

- Master in medical education
- Master in health professional education
- Master of teacher education
- Master in surgical education
- Master in medical and dental education
- Master of clinical education
- Master in health and medical education
- Master in academic medicine
- Master in higher education with specialization in health professional education [7].

Curriculum planning is one of the main areas of education that plays a key role in the optimal training of human resources. A curriculum is the outline of an educational activity, in other words, the agenda of education. Curriculum development is a process that is used to identify needs and specific objectives of education, identify and organize the content of education, select teaching methods and provide materials needed to evaluate education and students [6].

If the curriculum is not rational and realistic, it not only will fail to produce the desired results, it may also lead to irreparable negative impacts [8, 9]. Therefore, care should be taken to avoid any carelessness and waste of resources, and methods that ensure to its quality, i.e. determining and managing the curriculum elements should be considered [9]. Since curriculum evaluation is a part of the educational system and one of the four essential components of any curriculum, addressing the shortcomings will improve a curriculum [1, 4, 9, 10].

One of the logical methods in the field of curriculum modernization is to conduct comparative studies, which by identifying the differences or similarities of the curriculum, enables solve educational problems and issues, and demonstrates a set of factors and effective contexts contributing to successes and the failures of the educational system. In addition, it is very helpful to observe creative examples and provide resources to facilitate the discovery and selection of required innovations [11].

Considering the foregoing and that there are few studies on curricula and now the number of universities that offer this education has increased, the aim of the present study is to investigate the status of the medical education curriculum in the studied universities and to provide practical suggestions to improve its status in Iran by identifying the possible shortcomings.

## Methods

This is an applied descriptive cross-sectional and comparative research. Data collection was carried out by electronic search on the website of the universities. Each of these selected curricula, which was the newest curriculum of the studied universities at the time of research, was translated into fluent Persian and studied in detail. The famous Polish Bereday model was used in the present study. This model is an absolute and abstract method of comparative study methods that identifies four stages of description, interpretation, juxtaposition, and comparison in comparative studies [12, 13].

In the description stage, research phenomena are prepared for review and critique at the next stage on the basis of evidence and information, note-taking and sufficient findings for review and critique in the next stage. In the interpretation stage, the described data is checked and analyzed, and in the juxtaposition stage, the data prepared in the previous two stages is categorized and put together in order to create a framework for comparing similarities and differences. In the comparison stage, the research problem is investigated and compared according to the details of similarities, differences and responses.

According to this model, first the required data about the medical education curriculum was collected from reliable sources and carefully studied and analyzed. This data was classified into tables and similarities and differences between all the studied universities in terms of the elements of the curriculum was determined. Finally, based on these similarities and differences, practical suggestions were prepared to improve each element of the Iranian curriculum.

The collected data were analyzed by Bereday method. In addition to thinking and reasoning, the similarities and differences of the texts were considered. In the first stage of the present study, the collected data about the studied countries were briefly compared and analyzed in comparative tables. Then, according to the similarities and differences, which are specified in the comparative tables, the specifications of the curriculum in master degree of medical education were obtained.

The study population includes the countries of the world and the documents of the curriculum in master degree of medical education. The study population included 76 universities that offered a curriculum in master degree of medical education. Inclusion criteria included presentation of curriculum in master degree of medical education, the presentation of the curriculum in Persian or English and the availability of the curriculum. Exclusion criteria also included universities with only in-person curricula and universities with non-Persian and English language curricula. Also, considering that a comparative study was conducted by Karimi Moneghi (2014) between the universities of Maastricht, Dundee, Calgary and Iran in the same field, these universities were also excluded from the study [6]. According to the inclusion criteria, the samples of the present study included seven selected universities including Kebangsaan University in Asia, University of Toronto in Canada, University of Michigan in America, University of Bern and Imperial College in Europe, Monash University in Australia and Iranian universities.

Universities were selected at a 1/10 ratio from each classified area using quota sampling. Institutions that offered curriculum in master degree of medical education were selected in each region using the World Health Organization (WHO) classification based on QS World University Rankings (2020) [14]. Institutions whose curriculum is presented only in person were not included in the present study. Also, because there were no English-language curricula at two top German universities in Europe, we had to choose a third university. Also, since curriculum information was not available at Australian universities, New South Wales and Sydney, the next ranked university, Monash, was investigated (Table 1).

**Table 1:** Selected universities

**Region according to WHO classification**	**Number (Percentage) of universities with a master** **’** **s degree in medical education**	**Selected University** **(Based on 2020 QS ranking)**
Europe	**33 (43%)** - 20 universities in the UK -13 universities in Europe	- Imperial College in the UK - University of Bern in Europe except United Kingdom
United States	**25 (26%)** -15 universities in the United States - 5 universities in Canada - 5 universities in Latin America	- Toronto in Canada - Michigan in the United States
Asia	**13 (17%)** - Seven universities in Iran - Two universities in Malaysia -One University in Bangladesh -One University in India -One University in Indonesia -One University in Thailand	- Kebangsaan in Malaysia - Iran
Middle East	**4 (5%)** - Two universities in Pakistan -One University in Egypt -One University in Saudi Arabia	-
Australia	**4 (5%)**	- Monash
South Africa	**2 (3%)**	-

The data collection instrument included a researcher-made checklist, which was used to extract the relevant information available on the website of selected universities. This checklist was consisted of eight items, which included course title, course length, mission, vision, goals, admission process, teaching methods (online, In-person and both), educational strategies and teaching methods and student assessment methods, which were compared in selected universities.

In order to determine the validity of data collection instrument, the content validity method was used. For this purpose, to prepare a data collection checklist, first the characteristics of the curriculum in master degree of medical education was prepared based on a curriculum component, and then the necessary corrections were made based on the expert opinion. To determine the reliability of the data collection instrument, the inter-rater reliability was used and confirmed (*r* = 0.89). Also, data extraction has been carried out by the research team to increase the accuracy and credibility of the extracted data.

It should be noted that due to the large data, the results of the description and interpretation stages of the Bereday model are presented in the Results Section and the results of juxtaposition and comparison stages in the Discussion section.

## Results

Samples of the present study included seven selected universities including Kebangsaan University in Asia, University of Toronto in Canada, University of Michigan in the United States, University of Bern and Imperial College in Europe, Monash University in Australia and Iranian universities. The following results are the result of a detailed study, description and interpretation of the curriculum in master degree of medical education.

1. Universities of Iran:
  Course Name: Master of Medical Education
  Course length: 3 years
  Mission:
    The main mission of medical education is to train competent, efficient and responsible human resources that provide their information and capabilities to the officials of the educational system and society when accepting educational policies and strategies, developing curricula at different levels, educational methods and techniques, managing educational systems, student assessment, evaluation of educational systems, transferring appropriate educational technologies. They also play a role in improving the education status of the universities by researching educational fields and then identifying strengths and weaknesses and providing appropriate solutions.
  Vision:
    In the next ten years, Iran will be recognized as a model in this field in terms of indicators and standards of education and research in the region.
  Goals:
    Graduates of this course will be able to:
     ◦ Prepare and guide lesson plans and educational guidelines for use in the classroom, laboratory, and clinical areas.
     ◦ Assist educational groups in revising curricula
     ◦ Actively participate in the formulation of educational policies and strategies of universities and departments.
     ◦ Use educational models, methods and techniques in various educational fields.
     ◦ Design and apply appropriate educational evaluation methods.
     ◦ Design and implement educational research in order to identify educational problems and provide appropriate solutions.
     ◦ Be familiar with the latest methods and techniques of student assessment and apply them.
     ◦ Be familiar with educational management techniques and apply them in educational fields.
  Terms and conditions for student admission:
    The entrance exam is passed in accordance with the rules and regulations of the Ministry of Health, Treatment and Medical Education. Incoming students are selected in order of priority from the following groups:
     ◦ Faculty members of medical sciences departments of universities and medical schools across Iran
     ◦ Faculty members of medical sciences departments of Islamic Azad University
     ◦ Persons who have a master’s degree in medical sciences.
     ◦ Persons who have general doctorate degrees in medicine, pharmacy and dentistry, clinical P.H.D. and clinical subspecialty.
  Type of education: In-person and online.
  Educational strategies and teaching methods:
     ✓ Educational strategies:
      - A combination of teacher-and student- centered approaches
      - Problem-solving-based nature
      - A combination of mandatory and optional topics
      - Based on classroom and field work
      - Based on professional tasks
     ✓ Training methods and techniques in this curriculum:
      - Mass teaching methods: such as conference presentation
      - Using techniques and methods of discussion in small groups such as: brain storming, buzz group, snow balling, workshop, etc.
      - Demonstrating clinical educational methods such as morning reporting, round, and outpatient training
      - Electronic education such as: teleconference, tele-communication, tele-consultation.
      - Online and simulation methods such as: role playing, SP, using skill, etc.
      - Self learning
      - Other methods and techniques according to educational goals
  Assessment:
    Assessment methods are used to assessment students, preferably new methods, in a combined and continuous form, as mentioned in each lesson in the course syllabus. Assessment is carried out both during and at the end of the semester [15].

Overall, in the curriculum of Iranian universities, are expected students to be able to treat professors; students, colleagues and patients, if any, with respect, observe ethical considerations when criticizing curricula, observe ethical considerations when conducting educational research, feel committed and responsible when maintaining the educational resources and equipment and follow the rules of the dressing code. The expected competencies for the graduates are the characteristics of this curriculum, which include: Establishing effective professional communication, designing various curricula, both traditional and innovative, designing various educational evaluations and related processes, basic skills in designing educational and multimedia materials, basic skills in effective teaching, criticize processes, texts and educational materials, basic skills in educational research, educational counseling, educational leadership and management, and basic English language skills.

2. University of Toronto:
  Course Name: Health practitioner teacher education
  Course length: Not mentioned.
  Mission:
    The University of Toronto is committed to nurturing academic communities that thrive on equity, learning, and scholarly membership, with rigorous human rights advocacy and a strong commitment to equal opportunity, justice, and equality.
    To engage in innovative research and education programs and service, all aimed at bringing the best science to the creation of the trans-disciplinary approaches, systems, and professionals needed to optimize individual as well as population health in the sustainable health system of the future.
  Vision:
    We will be an internationally recognized academic unit dedicated to developing, testing, evaluating and teaching approaches to integrating primary care1, preventive medicine & public health.
  Goals:
    Pursue the synthesis of theory, methods and practice through:
     ◦ The development of “Clinical Public Health” (the integration of primary care, preventive medicine & public health) activities within the university, associated hospitals and community health centers, and associated public health agencies, aimed at fostering collaborative research, training and knowledge translation programs.
     ◦ The development of discrete “Participatory” opportunities for testing and evaluating “Clinical Public Health” system-wide approaches to optimizing the health of targeted population units through innovative improvements in the integration of primary care, preventive medicine & public health.
     ◦ Use of the current Determinants of Community Health (DOCH) medical school curriculum as a platform for developing a robust inter-professional education and training (IPE&T) component of the standard medical education curriculum that embraces the integration of primary care, preventive medicine & public health ethos of “Clinical Public Health”.
     ◦ The development of innovative training of all public health students in basic clinical practice and health systems and its evolution into a robust inter-professional education and training (IPE&T) component of the standard public health curriculum.
     ◦ Exploration of the potential development of innovative IPE&T programs for nursing, pharmacy, social work, kinesiology and other health science training programs.
     ◦ The training of clinical public health leaders through
     ◦ The existing robust Residency training program in Preventive Medicine & Public Health.
     ◦ The development of innovative MD MPH, RN MPH, and other such combined degree program.
     ◦ The development of an MD PhD track in public health sciences research in the medical scientist training program Provide leadership in the academic and professional communities in the area of “Clinical Public Health.”
  Terms and conditions of admissions:
    MSc students are admitted under the General Regulations of the School of Graduate Studies (SGS) and should hold an appropriate bachelor’s degree or its equivalent from a recognized university with at least a mid-B average in final year of the degree, or in the last 5.0 full course equivalents completed at a senior level. Proof of English Language Proficiency is required.
  Type of education: In-person and online.
  Educational strategies and teaching methods:
     ◦ Continuing planning
     ◦ Rethinking assessment
     ◦ Classroom management
     ◦ First class strategies
     ◦ Tone adjustment
     ◦ Teaching a large classroom
     ◦ Inclusive teaching
     ◦ Teacher-student supportive interaction
     ◦ Academic integrity and the role of the teacher
     ◦ Tips for potential risks in online teaching learning
  Assessment: No information was obtained in this regard [16, 17].

Overall, the curriculum encourages students to take optional courses to enhance their learning experience or perform a focused study on specific topics. Developing, evaluating, supporting technology-enabled learning/teaching, evidence-based learning, and community engagement in course design to create data analysis dashboards, provide a variety of projects that support their strategic priorities. Rethinking is an essential component of all experiential learning activities. It is a process that allows students to focus on what they have learned in the context of experiential learning and the integration of this disciplinary knowledge.

3. Monash University:
  Course Name: Health professional education.
  Course length: 1 year (Maximum 3 years)
  Mission:
    We will strive to achieve excellence in education to ensure our students serve the good of their communities. Curriculum will be infused with internationalism and enterprise to prepare our graduates for their futures in a multi-skillset workforce. We will be inclusive – embracing the talented independent of their social or economic circumstances.
  Vision:
    Monash is working to deliver education of exceptional quality on an unprecedented scale. At the heart of our new vision for education is a commitment to an outstanding student experience that is underpinned by the four pillars of Focus Monash.
  Goals:
    Monash University trains graduates with the following traits:
     1. They are effective and accountable global citizens:
      a. They are involved in the international world.
      b. They demonstrate intercultural competency.
      c. They demonstrate moral values.
     2. They are creative and critical scientists who:
      a. Provide innovative solutions to problems.
      b. Use research skills to deal with various challenges.
      c. Communicate effectively, sensitively and intelligently.
  Terms and conditions of student admission:
     ◦ Bachelor’s degree (or equivalent) in health-related fields
     ◦ English language certificate
     ◦ Minimum requirements of Monash University
  Type of education: In-person and online
  Educational strategies and teaching methods: No information was obtained in this regard.
  Assessment methods: No information was obtained in this regard [17].

Overall, the university offers courses that are consistent with the university’s educational and strategic priorities and academic standards. The graduates’ characteristics also match with the Australian competency framework, which is based on the Bologna Process. Moreover, the educational priorities of this curriculum include future-oriented education, empowerment of social mobility, support of individuals and natural knowledge, entry of rich experiences and success through teaching innovative and flexible learning.

4. Kebangsaan University:
  Course Name: Master of Medical Education
  Course length: 2 years
  Mission:
    Provide quality and up-to-date education through the application of research results to produce ethical and competent health professionals to improve the health and well-being of society.
  Vision:
    To be a regional educational excellence center in the field of health sciences by providing quality and up-to-date knowledge in the field of education, research and development as well as community services while supporting national identity.
  Goals:
     ◦ To achieve and maintain world-class quality education and nature individual excellence within national model of national identity.
     ◦ To strengthen research culture, widen, and reinforce global strategic partnerships in line with research university status.
     ◦ To promote social responsibility in health sciences for the benefit of society.
     ◦ To achieve and sustain an efficient delivery system to support the strategic plan of faculty members.
  Terms and conditions of student admission:
     ◦ Checking resume and taking the entrance exam:
     ◦ Medical students or those who have a bachelor’s degree from a reputable institution with a GPA higher than 2.75
     ◦ Non-medical graduates should submit a letter indicating that they work in a medical or other health education institution.
     ◦ Participating in the entrance exam that covers the academic potential exam, the English and Indonesian language proficiency test.
  Type of education: In-person and online.
  Educational strategies and teaching methods:
     ◦ Lecture
     ◦ Discussion
     ◦ Seminar
     ◦ Field work
  Assessment methods: No information was obtained in this regard [18].

Overall, to achieve national identity, the university’s curriculum seeks to train graduates who are a scientist and up to date in their related fields, competent, confidante and able to compete globally, have analytical and critical thinking and able in conducting research, emphasize lifelong learning, have effective communication skills, demonstrate high moral values when taking on social responsibilities, ethical, committed and professional, and have the ability to work in a team, manage, execute, a leader and an entrepreneur in the health sciences.

5. University of Michigan:
  Course Name: Health professions education- master of arts
  Course length: 2 years (Maximum 5 years)
  Mission:
    The mission of this international center is to provide services and programs to the diverse community at the University of Michigan so that it can provide services to the international population, FACILITATE intercultural and international education, and FOSTER a global campus community.
  Vision:
    Fostering a global campus through mutual respect and understanding is the vision of the university. All members of societies transform and grow together. We strive for inclusive, intercultural excellence where each individual differentiate value from shared experiences.
  Terms and conditions of student admission:
    Through the resume and some evidence, including the applicant’s previous educational record, work experience in the training position and the expression of professional goals that are consistent with the mental focus and online format of the curriculum, are also examined.
    Admission applications are reviewed by faculty members of the College of Education in order to examine documents of adequate preparation for advanced professional study at the master’s level and to assess the likelihood of success in online learning. Some documents, including the applicant’s previous educational record, work experience in the training position, and the expression of professional goals that are consistent with the mental focus and online format of the curriculum, is also investigated.
  Educational methods: In-person and online
  Educational strategies and teaching methods:
     ◦ Competency-based training
     ◦ It is individualized according to the learner’ position.
     ◦ It comes along with adaptive individual mentoring.
  Assessment methods: No information was obtained in this regard [19].

Overall, the university offers a competency-based curriculum. The curriculum is individualized according to the learner’s position and comes with individual adaptive mentoring. The issue of correct and reliable assessment of learner competency is very important in this curriculum. The assessment method has two basic components: the independent assessment committee and the learner repository. The learner submits competency-related documents that are assessed by three independent assessors. The assessment results are submitted to the assessment committee. The learner receives feedback on the submission and activities may be required to related competency if needed. In this curriculum, monitoring is carried out individually and all faculty mentors are trained and experienced in medical education.

6. Imperial College University
  Course Name: Surgical education
  Course length: (Table 2)

**Table 2:** Course length in Imperial College University

**Total credit**		**Study style**	**Course length (month)**	**Certificate**
**CATS** ^a^	**ECTS** ^b^			
60	30	Part-time	12	Online PG Certificate
120	60	Part-time	24	Combined PG diploma
180	90	Part-time	36	Combined MEd

^a^ The national credit accumulation and transfer scheme

^b^ European credit transfer and accumulation system
  Curriculum presentation: Online
  Mission:
    Imperial College embodies and delivers world class scholarship, education and research in science, engineering, medicine and business, with particular regard to their application in industry, commerce and healthcare.
    We foster multidisciplinary working internally and collaborate widely externally.
  Vision:
    ◦ Maintaining the institute for education and scientific research
    ◦ Increasing the quality, depth and breadth of research capabilities to address the current and future problems and challenges.
    ◦ Training next generation of researchers, scientists and academicians.
    ◦ Providing education to students around the world so that they can achieve the knowledge and skills they require to pursue their ambitions.
    ◦ Creating social and economic impact by translating our work into practice worldwide
    ◦ Engaging with the world and communicate the importance and benefits of science for society.
  Terms and conditions of student admission:
    It is through interviews / admission tests and applications are typically evaluated by curriculum leaders and decisions are made based on the degree of engagement in the training as demonstrated through: experience, letters of arbitration and personal statements.
  Educational method: In-person and online
  Educational strategies and teaching methods:
    ◦ Workshop
    ◦ Journal club
    ◦ Seminar
    ◦ Lecture
    ◦ Flipped classroom
    ◦ Simulation
    ◦ Role-play
  Assessment method:
    This curriculum uses constructive and final assessment methods in surgery education, that are designed to develop and deepen your knowledge and practice as a surgery instructor, and assessment opportunities are selected to rethink the nature of the module and the learning outcomes of the curriculum (Table 3).

**Table 3:** Assessment methods in Imperial College University

	**PG Cert**	**Diploma**	**MEd**
Course work	75%	77%	70%
Practical	25%	23%	30%
Written exams	0%	0%	0%
    Assignments are designed based on the activities performed by the surgery instructor (design of learning teaching activities, course / session design, development of assessment tools and portfolio, and implementation of educational research) [20].

Overall, the university curriculum is offered part-time and in a modular manner, with the aim of challenging the thinking of surgical instructors and developing their practice, developing a functional and theoretical understanding of surgical instruction, and developing the judgment needed to be innovative in the growing and important field of surgery and its curriculum structure.

7. University of Bern
  Course Name: Master of Medical Education
  Course length: 2 years (Maximum 4 years)
  Mission:
    To achieve the goals of participating in the development of society and the economy, the University of Bern aims to increase the attractiveness of the Bern sector and help strengthen efforts for sustainable development.
  Vision:
    Knowledge is one of the most important resources in our society that will become more important in the future. Knowledge is the key to solving social problems and overcoming global challenges. Universities make an indispensable contribution to the evolution and imparting of knowledge, and this task will become increasingly important.
    The University of Bern wants to procure the competences required to acquire knowledge and to use that knowledge. With this aim in mind, the University of Bern conducts excellent research and teaching and offers high-quality advanced training and services. The University of Bern bases it’s disciplinary, interdisciplinary and transdisciplinary focuses on academic interests and societal needs.
    The university aspires to be the most important institution in the region that produces independent knowledge and promotes sustainable value for the Bern economy. The university will make a major contribution to increase Bern’s appeal as a center of economic innovation and attractive working conditions.
  Goals:
     ◦ Training competent leaders in medical education
     ◦ Benefiting from teaching knowledge based on evidence and teaching methods
     ◦ Supporting the development of new teaching projects and curricula
     ◦ Improving leadership skills in the medical sector
     ◦ Benefiting from academic knowledge at the level of medical education
     ◦ Communicating with national and international experts
     ◦ Building a network between participants and promoting a participatory and active learning environment
  Terms and conditions of student admission:
    The admission decision is made by the curriculum managers based on the documentation and the written request. The decision on admission or non- admission is usually announced 6-8 weeks after the application. The minimum requirements for a master’s degree include:
     ◦ Successful completion of at least nine courses (in five weeks) including nine core courses.
     ◦ Writing at least six assignments after the course (3 core courses).
     ◦ Performing a project with a minimum score of E.
     ◦ Writing a master’s thesis with a minimum score of E.
     ◦ Acquisition of 60 ECTS credits.
  Educational method: In-person and online.
  Educational strategies and teaching methods: No information was obtained in this regard.
  Assessment method: No information was obtained in this regard [21].

Overall, the university’s curriculum includes 12 one-week courses. To receive a master’s degree, it is necessary to attend at least nine weeks of the course, six of which must be core courses. Depending on the content, the modules cover relatively comprehensive topics. The focus is on content such as curriculum planning, cognitive psychology, assessment, leadership and educational research. More than 90% of the courses are offered in English, with the exception of the second week course which relates to the communication field and is offered in German.

## Discussion

The information obtained from the juxtaposition and comparison of medical education curricula in seven selected universities in the present study is shown in Table 4.

**Table 4:** Comparison of the curricula in master degree of medical education in the studied universities

**Selected University**	**Course length**	**Student admission**	**Values / mission and vision**	**Goals**	**Expected Outcomes / Competency**	**Educational strategies and methods**	**Student assessment**
Universities of Iran	3 Year	Passing the entrance exam is in accordance with the rules and regulations of the Ministry of Health, Treatment and Medical Education. Incoming students are selected in order of priority	**Values and beliefs:** -Divine and spiritual principles in the education of human beings -Rich culture of Islamic-Iranian education as much as possible - Promoting education in medical sciences in order to promote community health -Applying the principles of professional and Islamic ethics in all affairs -Responsive education - Individual growth of learners - Variety of educational methods -Global standards - Nurturing creativity in learners **Mission:** The main mission of medical education is to train competent, efficient and responsible human resources that provide their information and capabilities to the officials of the educational system and society when accepting educational policies and strategies, developing curricula at different levels, educational methods and techniques, managing educational systems, student assessment, evaluation of educational systems, transferring appropriate educational technologies. They also play a role in improving the education status of the universities by researching educational fields and then identifying strengths and weaknesses and providing appropriate solutions **Vision:** In the next ten years, Iran will be recognized as a model in this field in terms of indices and standards of education and research in the region	Graduates of this course will be able to: -Prepare and guide lesson plans and educational guidelines for use in the classroom, laboratory, and clinical areas - Assist educational groups in modifying curricula - Actively participate in the formulation of educational policies and strategies of universities and departments - Use educational models, methods and techniques in various educational fields -Design and apply appropriate educational assessment methods - Design and implement educational research in order to identify educational problems and provide appropriate solutions - Be familiar with the latest methods and techniques of student assessment and apply them - Be familiar with educational management techniques and apply them in educational fields	-Establishing effective professional communication -Designing various educational curricula, both traditional and innovative -Designing various educational assessment s and related processes -Basic skills in designing educational and multimedia materials - Basic skills in effective teaching -Critique of processes, texts and educational materials - Basic skills in educational research - Educational counseling - Educational leadership and management - Basic English language skills	**Strategies:** This curriculum has been developed based on the following strategies: - A combination of teacher-and student- centered approaches - Problem-solving-based nature - A combination of mandatory and optional topics - Based on classroom and field work - Based on professional tasks -Teaching methods and techniques (learning/teaching methods) **Teaching methods:** -Participative teaching methods, such as conference presentation - Using techniques and methods of discussion in small groups such as: brain storming, buzz group, snow balling, workshop, etc -Demonstrating clinical educational methods such as morning reporting, round, and outpatient education - Electronic education such as: teleconference, tele-communication, tele-consultation - Online and simulation methods such as: role playing, SP, using skill, etc - Self-learning - Other methods and techniques according to educational goals	Assessment methods are used to assess students, preferably new methods, in a combined and continuous form, as mentioned in each lesson in the course section. Assessment is carried out both during and at the end of the semester
University of Toronto	It has not been stated	MSc students are admitted according to the general rules of graduate studies and must have a bachelor’s degree or its equivalent from a famous university, or at least a B average in the last year. English language proficiency level is required	The University of Toronto is committed to nurturing academic communities that thrive on equity, learning, and scholarly membership, with rigorous human rights advocacy and a strong commitment to equal opportunity, justice, and equality. In educational, research, and service curricula, all goals are to achieve the best science in creating interdisciplinary and systemic approaches to empowering individuals (for example, in community health in a resilient future health system). University Vision: They should be recognized internationally as an academic unit specializing in the development, testing and assessment of teaching approaches for integrating primary care, preventive medicine and general health	Synthesis of theory, method and practice through: - Development of “clinical general health” activities (integration of primary care, preventive medicine and general health) within the university, as well as hospitals and social health centers and public health agencies, with the aim of fostering collaborative research, training and knowledge translation plan -Separate development of “participatory” opportunities to test and evaluate approaches to the breadth of the “clinical general health” system to optimize the health of target population units through innovative improvements in the integration of primary care, preventive medicine, and general health -Using the curriculum determinants of the Medical School of Social Health as a platform for the development of interprofessional education components of the standard medical education curriculum that integrates primary care, preventive medicine, and “public health” social behaviors and beliefs - Development of innovative education of all public health students in basic health systems and clinical practice and development into components of standard interprofessional education in the standard public health curriculum - Identifying the potential development of innovative IPE & T curricula for pharmacy nursing, social work, mechanism principles, and other health science education curricula - Clinical general health education through the robust residency education curriculum in general health and preventive medicine - Development of MD MPH RN MPH and other combined curricula - Development of MD PhD in public health science research in medical education curriculum, providing leadership in professional and academic communities at the levels of clinical general health	It has not been stated	**Teaching strategies:** - Continuing planning - Rethinking assessment - Classroom management - First class strategies - Tone adjustment - Teaching a large classroom - Inclusive teaching - Supportive teacher-student interaction - Academic integrity and the role of the teacher - Tips for potential risks in online teaching learning	It has not been stated
University of Michigan	2 years (Maximum 5 years)	Admission applications are investigated by faculty members of the College of Education in order to examine documents of adequate preparation for advanced professional study at the master’s level and to assess the likelihood of success in online learning Through resume: Some documents, including the applicant’s previous educational record, work experience in the educational position, and the expression of professional goals that are consistent with the mental focus and online format of the curriculum, is also investigated	**Mission:** The mission of this international center is to provide services and curricula to diverse communities at the University of Michigan so that it can provide services to the international community **Vision:** Cultivating a global campus through mutual attention and understanding is the vision of the university. All members of societies evolve and grow together. We strive for universal intercultural excellence where each individual differentiate value from shared experiences	-Link behavioral and operational decisions with strategic goals - Provide exceptional and extraordinary services - Foster a learning, growth and teamwork environment - Continuous review of policies and procedures with the mentality of moving towards improvement - Communicate with the society - Innovation and use of technology	Five areas and twelve competencies: **Area 1: Teaching–learning theory** 1.Education theory 2.The context of health education **Area 2: Teaching performance** 3.Educational methods 4.Curriculum planning 5. Educational community **Area 3. Assessment and Evaluation** 6. Assessment 7. Evaluation **Area 4. Research and scholarship** 8. Research methods 9. Educational scholarship **Area 5. Leadership** 10.Individual leadership skills 11. Leadership theory 12.Organizational leadership	-Competency-based education -It is individualized according to the learner’ situation -It comes with adaptive individual mentoring	The issue of correct and reliable assessment of learner competency is very important in this curriculum The assessment method consists of two basic components: -the independent assessment committee -the learner repository The learner submits competency-related documents that are assessed by three independent assessors. The assessment results are submitted to the assessment committee The learner receives feedback on the submission and activities may be required to related competency if needed In this curriculum, monitoring is carried out individually and all the faculty mentors in medical teaching are trained and experienced in guiding the learner in the EPAs EPAs include 12 competencies as tools for learning knowledge, skills and attitudes
University of Bern	2 years (Maximum 4 years)	Through resume: The admission decision is made by the curriculum managers based on the documents and the written request. The decision on admission or non-admission is usually announced 6–8 weeks after the application	**Mission:** To achieve the goals of participating in the development of society and the economy, the University of Bern aims to increase the attractiveness of the Bern sector and help strengthen efforts for sustainable development **Vision:** Attention is paid The University of Bern is summed up in three words, namely, knowledge generates value. The aim of the University of Bern is to acquire competency and knowledge and apply that knowledge. With this in mind, the University of Bern leads research and higher education and provides high quality services and education. At the University of Bern, with a focus on discipline and interdisciplinary approaches, much too academic interests and social needs	-Training competent leaders in medical education -Benefiting from teaching knowledge based on evidence and teaching methods -Supporting the development of new teaching projects and curricula - Improving leadership skills in the medical sector -Benefiting from academic knowledge at the level of medical education -Communicating with national and international experts - Building a network between participants and promoting a participatory and active learning environment	It has not been stated	**Educational strategy:** - Integration **Teaching methods:** - Classroom learning - Self-learning - Distance learning - Project presentation	It has not been stated
Imperial College University	3 years (Part-time)	Admission interview / Test: Typically, applications are evaluated by curriculum leaders and decisions are made based on their engagement in the education process, as shown by experience, letters of arbitration, and personal statements	**Mission:** Scholarship is responsible for providing world-class education and research with a special focus on their application in industry, commerce and healthcare in the fields of science, engineering, medicine and business. We cultivate interdisciplinary work **Vision:** - Maintaining the institute for education and scientific research - Increasing the quality, depth and breadth of research ability to solve current and future problems and challenges - Training next generation of researchers, scientists and academicians -Providing education to students around the world so that they can achieve the knowledge and skills they need to pursue their dreams - Creating social and economic impact by translating our research projects at the global level - Communicating with the world and determining the importance and benefits of science for society	- Challenging the thinking of surgical instructors and developing their practice - Developing a functional and theoretical understanding of surgical instruction - Developing the judgment needed to be innovative in the growing and important field of surgery and its curriculum structure	**PG certificate** In the end, they should be able to: -Evaluate contemporary institutional surgery structures, practices, frameworks and emerging policies considering stakeholder interests - Demonstrate practical training skills as a surgical instructor in a variety of contexts, including: learning, teaching, education, and assessment, including the design and assessment of training sessions, courses, and curricula -The ethical aspects of surgical education include the range of different learners, patients, and colleagues. Pay attention to their inclusion in their individual educational practice - Evaluate and develop reflection on their, others, and educational performance **PG Diploma** In addition to the outcomes of a PG certificate, they must be able to: -Apply and reflect on the theories, philosophies, and evidence available to promote surgical education commensurate with the range of suffering of individuals to national institutions and contexts -Critically critique the role and application of learning innovations in surgical education -Design creative solutions to challenges in contemporary surgery education by receiving training from other fields and functional areas -Demonstrate effective teaching verbal and written skills for surgical educational contexts -Synthesize recent knowledge about a field of surgical education with the aim of identifying areas for future research **MEd in surgical training** In addition to the above outcomes, they should also be able to: - Critically evaluate the use of educational research approaches in understanding the educational progress of surgery -Justify the use of educational philosophy and theories with an appropriate research question and research design -Produce an original small-scale surgical educational research project through a dissertation -Rethink their learning and development as a surgeon teacher and researcher	The overall workload includes teaching activities (In-person and / or online sessions) and Independent learning. Since the actual calling hours may be somewhat unclear, it is further specified how much time you need to devote to different activities at each level of the program In Imperial, each ECTS credit is equivalent to 25 h of study time (such as reading, writing, activities and lectures), at each stage of the program, 30 ECTS credits are offered, approximately 750 h of the total workload per year	MEd uses constructive and final assessment methods in surgery education, that are designed to develop and deepen their knowledge and practice as a surgery instructor, and assessment opportunities are selected to rethink the nature of the module and the learning outcomes of the curriculum Assignments are designed based on the activities performed by the surgery instructor (design of learning/teaching activities, course / session design, development of assessment tools and portfolio, and implementation of educational research) Constructive assessments are often developed and implemented in small groups, and constructive feedback is provided by the tutor and peers to develop topics such as presentation, teaching and writing skills. In the case of online modules, these assessments are carried out online and through the video conferencing platform Diploma and ME modules are held In-person or online on campus, and constructive assignments often end with final assignments Course works (constructive drafts) from the diploma literature review Project and the MEd thesis project are reviewed by the relevant supervisor Peer assessment enables you to reap the assumed learning benefits in this practice In practice, some assessment tasks are a combination of course work and practical components
Kebangsaan University	2 years	-Checking resume and taking the entrance exam: - Medical students or those who have a bachelor’s degree from a reputable institution with a GPA higher than 2.75 -Non-medical graduates should submit a letter indicating that they work in a medical or other health education institution -Participating in the entrance exam that covers the academic potential exam, the English and Indonesian language proficiency test	**Core values:** P- Prihatin (care) I-Integrity (Integrity) N-Nyalarasa (passion) K-Kreatif (creative) **Mission:** Provide quality and up-to-date education through the application of research results to produce ethical and competent health professionals to improve the health and well-being of society **Vision:** To be a regional educational excellence center in the field of health sciences by providing quality and up-to-date knowledge in the field of education, research and development as well as community services while supporting national identity	- Implementing higher education in the field of medical education, which is in line with the development of knowledge in medical education, both at the regional and global levels -Organizing research in high-quality medical education and printed results - Providing social services in the field of medical education based on the needs of the society - Establishing a partnership network with global and regional specialists in medical education at the level of education, research and social services - Training competent medical graduates who are ready to enter the labor market	Training of graduates who are equipped with a national identity that: 1. Be a scientist and be up to date in their related fields 2. Be competent and confidante and be able to compete globally 3. Have analytical and critical thinking and be able to conduct research 4. Emphasize lifelong learning 5. Have effective communication skills 6. Demonstrate high moral values when taking on social responsibilities 7. Be ethical, committed and professional 8. Have the ability of performing a team work, management, implementation, guidance and entrepreneurship in health sciences	It is facilitated for students to acquire competencies in the field of medical education management through various learning experiences such as lectures, discussions, seminars and fieldwork, in order to gain access to medical education management competencies based on relevant and up-to-date theories, especially in relation to curriculum design and implementation	It has not been stated
Monash University	1 year (Maximum 3 years)	-Bachelor’s degree (or equivalent) in health-related fields -English language certificate -Minimum requirements of Monash University	**Mission:** We will strive to achieve excellence in education to ensure our students serve the good of their communities. Curriculum will be infused with internationalism and enterprise to prepare our graduates for their futures in a multi-skillset workforce. We will be inclusive – embracing the talented independent of their social or economic circumstances **Vision:** Monash is working to deliver education of exceptional quality on an unprecedented scale. At the heart of our new vision for education is a commitment to an outstanding student experience that is underpinned by the four pillars of Focus Monash	-Express a personal philosophy of learner-centered teaching -Describe and apply the underlying learning theories of educational practice -Determine and apply the principles of curriculum design, the use of innovative educational approaches to achieve learning goals - Review contemporary teaching methods for a range of clinical contexts -Plan, design, implement, assess, and evaluate a curriculum -Describe and apply feedback strategies to support learning -Determine the role of mentoring and monitoring for safe clinical practice -Develop strategies for future learning as a health educator	Monash University trains graduates with the following traits: 1. They are effective and accountable global citizens: a. They are involved in the international world b. They demonstrate intercultural competency c. They demonstrate moral values 2. They are creative and critical scientists who: a. Provide innovative solutions to problems b. use research skills to deal with various challenges c. Communicate effectively, sensitively and intelligently	A very innovative approach to certification in continuing education, where half of the award degrees are completed with approved short courses and half with award units	It has not been stated

To the best of our knowledge, there has been no comparison of the curriculum in master degree of medical education, which focused on the topics studied in the present study. In addition to presenting the characteristics of the curriculum in master degree of medical education in Iran and selected countries, the present study showed the differences and similarities of this course among the top universities of different continents. But only one study compared the medical education curricula in the universities of Dundee, Calgary and Maastricht. The results of the above study showed that the universities of Dundee, Calgary and Maastricht focused on expressing the general characteristics of the curriculum clearly on medical sciences education. The mission and vision, values ​​and beliefs of the field are not observed in any of the studied curricula. The educational strategies used in Dundee and Maastricht Universities are explained by referring to the reason for their use as well manner of course presentation. All three universities also accepted undergraduate students, and there was a lot of emphasis on the research methods in education in the list of courses of all universities [6].

According to results of the present study, unlike other countries, the curriculum in master degree of medical education in Iran is offered in a centralized manner in eight universities of Tehran, Shahid Beheshti, Iran, Isfahan, Shiraz, Tabriz, Kerman and Mashhad.

Student’s admission in Iran is carried out through centralized exams, and therefore, most faculties do not have the option to select students and also criteria for student selection. The course length is between 1–3 years in all universities (depending on part-time / full-time) while most of the studied universities offer this field in a modular manner [15].

Before the Covid-19 pandemic, courses were offered in person or online, but, the course is now held online in all universities. At the end of the master’s degree, the dissertation is presented by the students of all universities, and if accepted, they will obtain a master’s degree.

## Conclusion

According to the previous extensive studies, the main proposal of the present study is to change and modify the competency-based curriculum of the Master of Medical Education since the recent trend has shifted to outcome-oriented education and competency-based curricula, universities have considered competencies and, have granted equivalent degrees to each person based on level of achievement of the desired competencies. For this purpose, the student assessment is based on the documents provided in the form of EPA activities and in the assessment committee. The course length is flexible and there are different presentation formats that each learner chooses the appropriate format based on his/her conditions.

## Data Availability

The datasets used and analyzed during the current study are available from the corresponding author on reasonable request.
